# The severity of developmental dysplasia of the hip does not correlate with the abnormality in pelvic incidence

**DOI:** 10.1186/s12891-020-03632-4

**Published:** 2020-09-21

**Authors:** Rongshan Cheng, Muyin Huang, Willem Alexander Kernkamp, Huiwu Li, Zhenan Zhu, Liao Wang, Tsung-Yuan Tsai

**Affiliations:** 1 School of Biomedical Engineering & Med-X Research Institute, Shanghai Jiao Tong University; Engineering Research Center of Digital Medicine and Clinical Translation, Ministry of Education, Shanghai, China; 2 Shanghai Key Laboratory of Orthopaedic Implants & Clinical Translation R&D Center of 3D Printing Technology, Department of Orthopaedic Surgery, Shanghai Ninth People’s Hospital, Shanghai Jiao Tong University School of Medicine, Shanghai, China; 3 Department of Biomedical Engineering, Case Western Reserve University, Cleveland, OH 44106 USA

**Keywords:** Developmental dysplasia of the hip, Pelvic incidence, Severity of DDH, Crowe classification

## Abstract

**Background:**

The purpose of this study was to investigate the association between the severity of Developmental dysplasia of the hip (DDH) and the abnormality in pelvic incidence (PI).

**Methods:**

This was a retrospective study analyzing 53 DDH patients and 53 non-DDH age-matched controls. Computed tomography images were used to construct three-dimensional pelvic model. The Crowe classification was used to classify the severity of DDH. The midpoint of the femoral head centers and sacral endplates were projected to the sagittal plane of the pelvis. The PI was defined as the angle between a line perpendicular to the sacral plate at its midpoint and a line connecting this point to the axis of the femoral heads. Independent sample t-tests were used to compare the differences between the PI of DDH group and the non-DDH controls group. Kendall’s coefficient of concordance was used to determine the correlation between the severity of DDH and PI*.*

**Results:**

Patients with DDH had a significantly (*p* = 0.041) higher PI than the non-DDH controls (DDH 47.6 ± 8.2°, normal 44.2 ± 8.8°). Crowe type I patients had a significantly (*p* = 0.038) higher PI (48.2 ± 7.6°) than the non-DDH controls. No significant difference between the PI in Crowe type II or III patients and the PI in non-DDH controls were found (Crowe type II, 50.2 ± 9.6°, *p* = 0.073; Crowe type III, 43.8 ± 7.2°, *p* = 0.930). No correlation was found between the severity of DDH and the PI (r = 0.091, *p* = 0.222).

**Conclusions:**

No correlation was found between the severity of DDH and the PI. The study confirmed that the PI in DDH (Crowe type I) group was higher than that of the non-DDH control group, while the PI does not correlate with the severity of DDH.

## Background

Developmental dysplasia of the hip (DDH) is characterized by a shallow, obliquely oriented acetabulum and is a known cause for secondary osteoarthritis (OA) of the hip [1]. Many patients with severe DDH present with an abnormal spinopelvic relation, which is coincident with low back pain (LBP) [2]. Due to the compelex deformity around hip, total hip arthroplasty (THA) in patients with OA secondary to DDH is reported with a higher rate of postoperative dislocations [3]. Wang et al. reported that DDH patients after THA experienced dislocation in 24 (2.93%) out of 820 hips [4]. The increased dislocation rates to DDH are most consistent with increased risk for post-operative THA dislocation patients that have a decreased pelvic incidence (PI) [2, 5, 6]. PI is one of the lumbosacral-pelvic anatomic parameters regulating the sagittal pelvic orientation and lumbar lordosis (LL) [7]. Hence, in DDH patients the increased or decreased PI may also cause the in higher rates of OA, LBP, and dislocations after THA. However, few studies have investigated this subject [2, 5, 6].

As an anatomical parameter, PI is unchanged when bone maturity has been reached and is unaffected by the pelvic position [8]. PI has been implicated in a number of disorders associated with the spine and hip, e.g., lumbar hyperlordosis [9], OA [6], LBP [10], hip dislocation [5], femoroacetabular impingement (FAI) [11, 12]. A similar observation of hip-spine syndrome has been discussed by Offierski and MacNab [13]. PI is associated with common secondary complications noted in DDH patients [1, 2]. However, PI is rarely considered during THA surgery in DDH patients. To reduce the dislocation rates and LBP, a better understanding of the relationship between PI in DDH patients may be useful for THA in patients with DDH. In addition, Boulay et al. reported that PI was an independent predictor of three-dimensional acetabular orientation [14]. Previous studies reported that DDH patients with decreased PI have a high rate of dislocation after THA, and LBP was relieved after THA in OA patients with high PI [5, 15]. Next, Imai et al. found that PI in DDH Crowe I patients was significantly higher by 4° than that in the non-DDH controls [16]. However, the association between the severity of DDH (i.e., Crowe I to IV) and the PI remains unknown.

We hypothesized that the severity of DDH was related to the abnormality in PI. The purpose of our study was to investigate (1) the differences between the PI in Crowe I-III DDH patients and non-DDH control subjects, and (2) the association between the severity of DDH and the abnormality in PI.

## Methods

### Patients

**Fig. 1:**
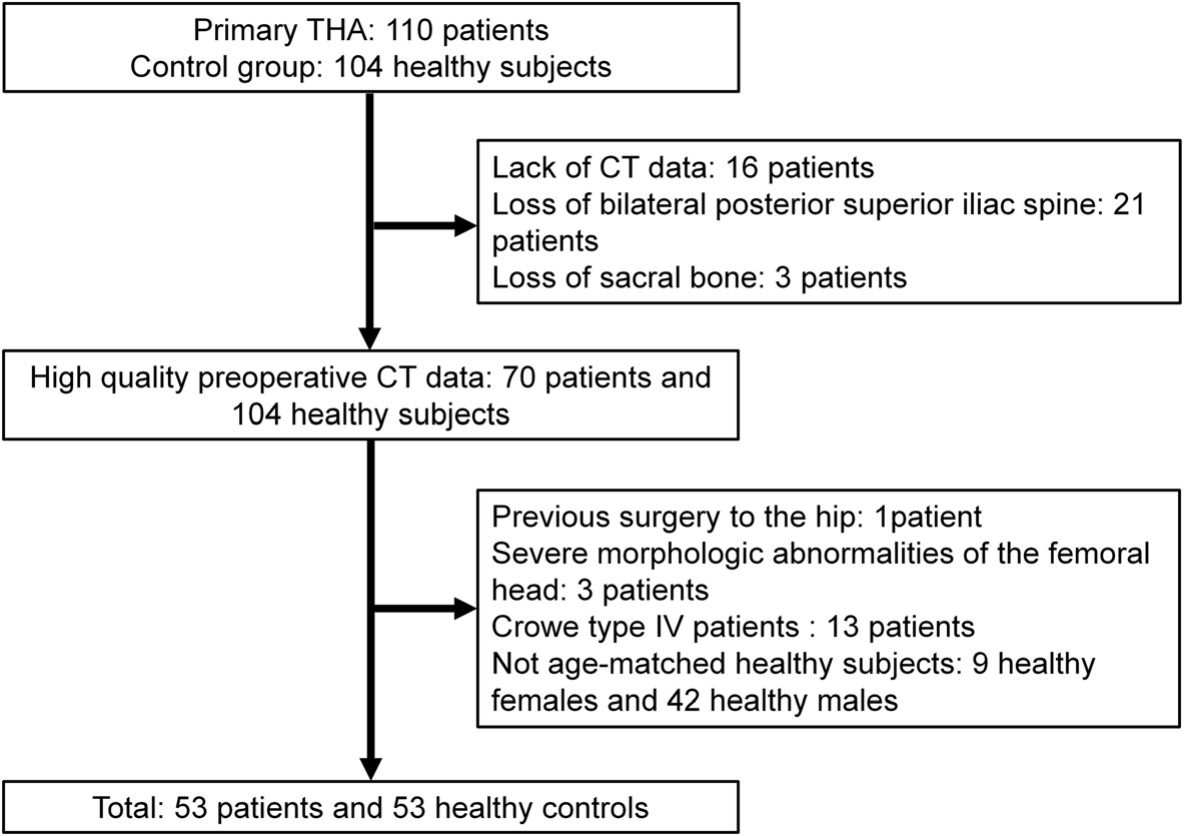
Flow chart diagram of patient selection

This retrospective study was approved by the independent ethics committee of Shanghai Ninth People’s Hospital (approval number 2016141). The preoperative computed tomography (CT) images of 110 DDH (i.e. the DDH group) patients who underwent THA between November 2007 and April 2017, and 104 high-resolution CT angiography images of the lower limbs which were diagnosed as a vascular disease between October 2011 and January 2015 (i.e., the control group) were retrieved. The inclusion criteria for patients diagnosed with DDH were: dysplasia with the lateral center-edge angle (LCEA) less than 20° [17]. The degree of dysplasia was measured from an AP radiograph according to the guideline of Crowe classification [18]. The exclusion criteria for patients diagnosed with DDH were: previous surgery to the hip or other hip diseases, severe morphologic abnormalities of the femoral head (the femoral head center could not be fitted due to the severe deformation of the femoral head), Crowe type IV patients lacking the femoral head and/or the sacral bone were excluded as the PI could not be measured according to the definition proposed by Legaye et al. [7]. The inclusion criteria for control group were: LCEA > 25°, and a sharp angle of < 45°. The exclusion criteria for the non-DDH control group were: hip disorders, abnormal or degenerative changes in the hip, and other hip symptoms. The condition of the non-THA side in DDH patients is normal, excluding hip diseases, severe morphologic abnormalities of the femoral head, or hip arthritic (Fig. 1). In total, 53 patients with DDH and 53 matched non-DDH controls were included in our study, 16 patients were excluded because of lack of postoperative CT scans and 13 patients because of crowe type IV patients. A detailed overview of the included patients can be found in Fig. 1. In the DDH group, 27 patients were Crowe I, 14 Crowe II, and 12 Crowe III. No significant differences were found between the baseline characteristics of the Crowe type I-III DDH patients and the non-DDH controls (Table 1).

**Table 1:** Comparison of characteristics in DDH (Crowe I-III) patients and non-DDH controls

Parameters	Non-DDH Controls, *N* = 53	Dysplastic, *N* = 27 Crow I	Dysplastic, *N* = 14 Crow II	Dysplastic, *N* = 12 Crow III	P †
Age* (yr)	55.5 ± 6.8	59.7 ± 9.1	55.6 ± 7.2	54.9 ± 11.8	0.181
Sex (no.)					0.090
Male	13	4	3	6	
Female	40	23	11	6	
Height* (cm)	159.8 ± 7.1	159.3 ± 4.9	161.1 ± 7.0	158.1 ± 3.4	0.813
Weight* (kg)	61.2 ± 5.9	59.1 ± 8.5	59.3 ± 7.4	58.6 ± 4.2	0.793
BMI* (kg/m2)	24.0 ± 2.2	23.3 ± 3.1	22.8 ± 2.5	23.4 ± 1.4	0.748

*Values express mean ± SD

† *P* values were obtained by ANOVA or chi-square test for comparisons in the DDH (Crowe I-III) patients and the non-DDH controls at 0.05 level

### Radiographic evaluations

**Fig. 2:**
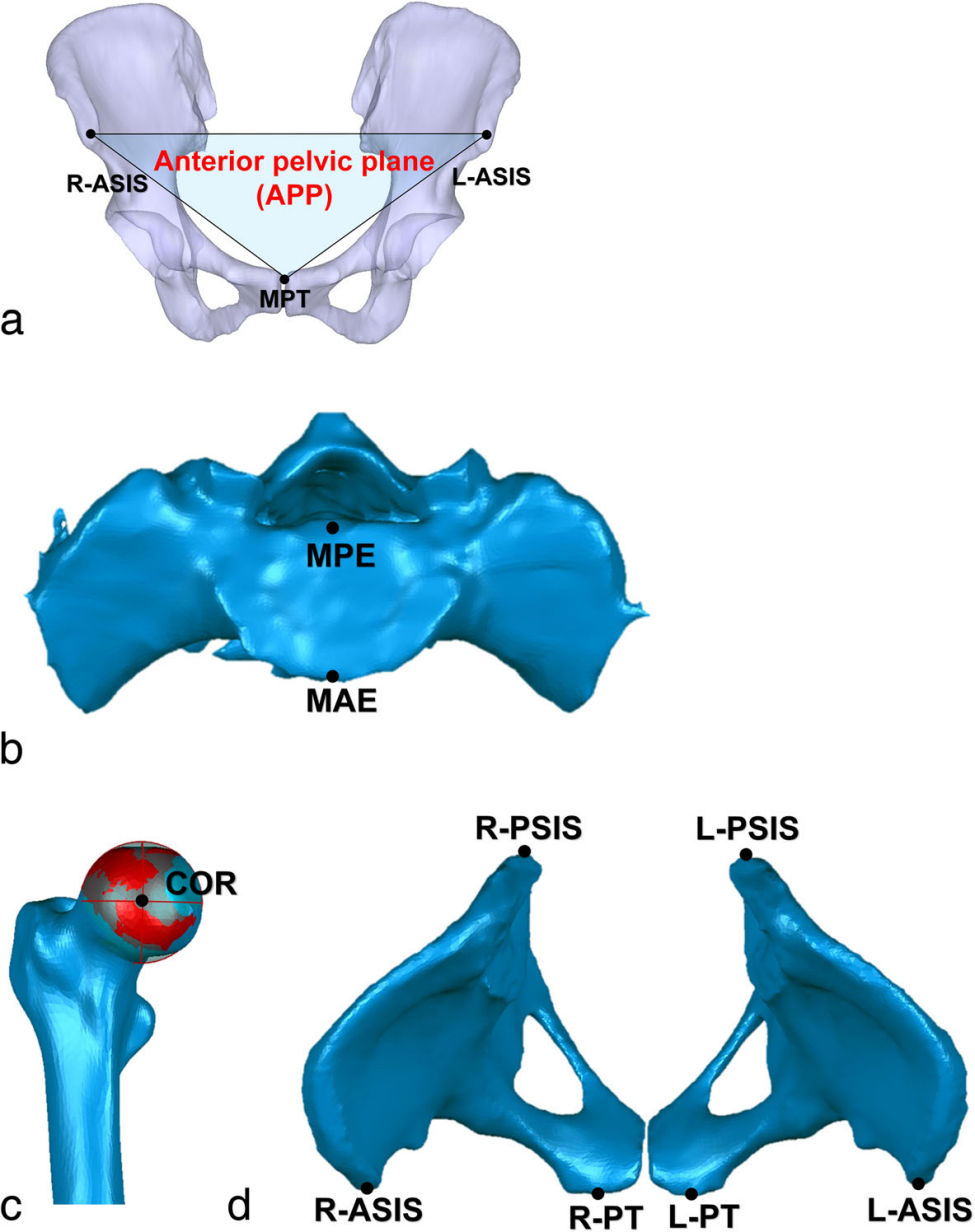
**a** The anterior pelvic plane (APP) was used for the pelvic coordinates, based on the anatomic bony landmarks, such as the right anterior superior iliac spine (R-ASIS), the left anterior superior iliac spine (L-ASIS) and the midpoint of the pubic tubercles (MPT). **b** The midpoint of the anterior edge (MAE) and the midpoint of the posterior edge (MPE) on the sacrum were defined. **c** The center of rotation (COR) was defined as the centroid of the best sphere (red-covered surface) to the surface of the femoral head (the mean standard deviation (STD) of the best-fit sphere of all femoral heads < 0.4 mm). **d** Bony landmarks of the pelvis including anterior-superior iliac spines (ASIS), pubic tubercles (PT) and posterior-superior iliac spine (PSIS) were digitized

CT scans of the DDH patients ranging from the fifth lumbar vertebra to the distal femur were collected using a 128-slices CT scanners (Somatom Definition Flash, Siemens Healthcare, Germany) with 1-mm slice thickness and an in-plane resolution of 0.98 mm. The CT scans of the non-DDH controls ranged from the fifth lumbar vertebra to distal femur were collected using a 64-slice CT scanner (Philips Medical Systems, Cleveland, Ohio, USA) with 2-mm slice thickness and an in-plane resolution of 0.68 mm. The CT images were then imported into the commercial software Amira (Amira, Thermo Fisher Scientific, Waltham, MA, USA) to construct three-dimensional (3D) surface models of the preoperative pelvis and femur. The 3D surface models of the sacral plate, pelvis, and femur were loaded into a self-written MATLAB script (MATLAB, The Mathworks Inc., Natick, MA) for subsequent data analysis (Fig. 2a). The midpoints of the anterior and posterior edges were defined as the points that divided the right and left halves of the sacral endplate in the coronal plane (Fig. 2b). The femoral head center of the patients with DDH and the non-DDH controls were determined as the centroid of a best fit 3D sphere to the surface of the femoral head (Fig. 2c). Locations of pelvic bony landmarks on bilateral anterior superior iliac spines (ASIS), posterior superior iliac spines (PSIS), and pubic tubercles (PT) were digitized on the 3D surface models to determine anatomic pelvic coordinate system (Fig. 2d).

### Measurement of the pelvic incidence

The anterior pelvic plane (APP) was used as a reference plan to establish the pelvic coordinate system of each subject [19], and the APP was corrected to zero degrees (Fig. 2a). The pelvic coordinate system origin is located halfway between the right and left ASISs with the medial-lateral (ML) axis running from the left to the right ASIS. The anterior-posterior (AP) axis, which passes through the origin and the mid-point of PSISs. The superior-inferior (SI) axis was the cross product of the AP and ML axes.

**Fig. 3:**
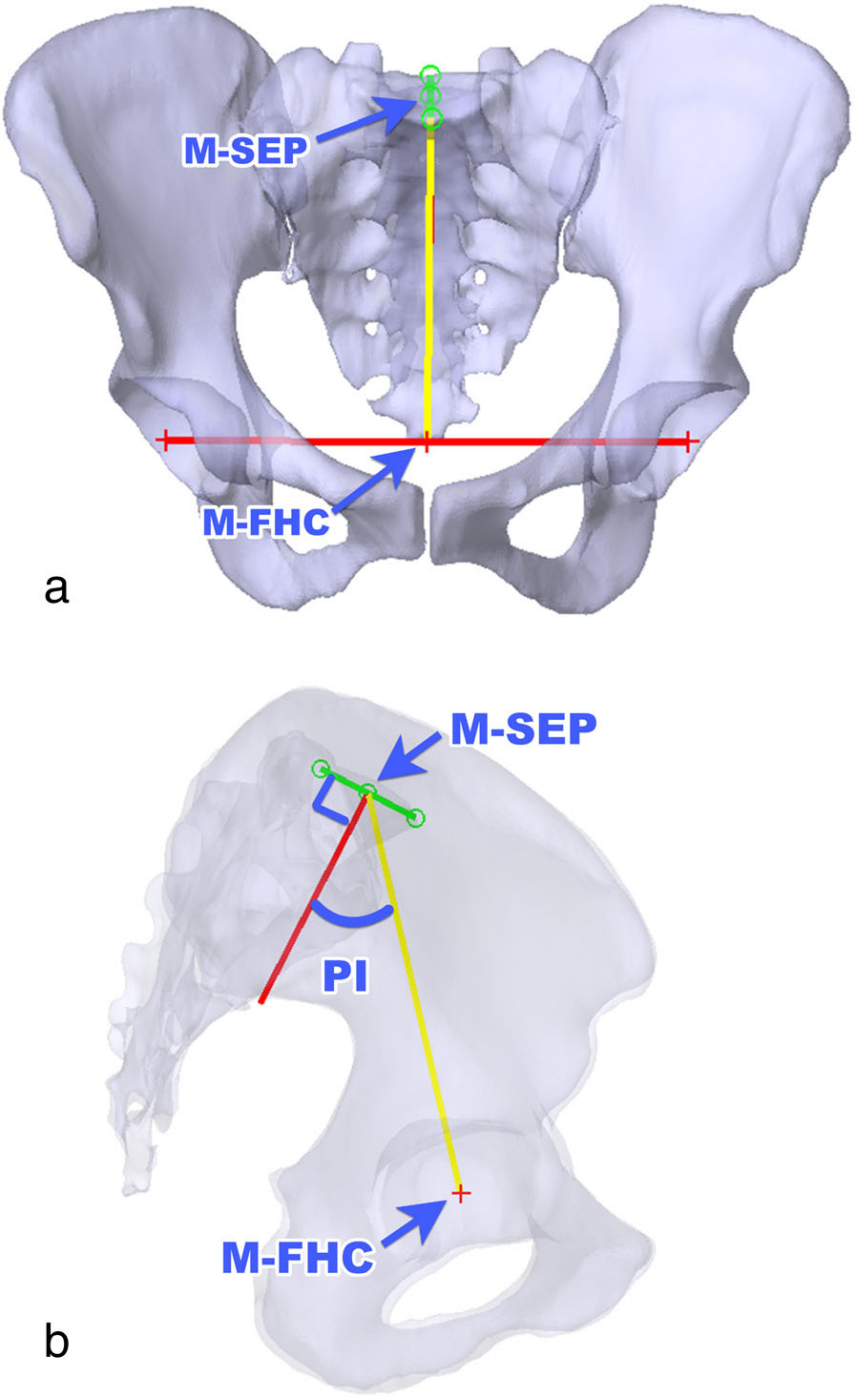
**a** M-SEP is defined as the midpoint of the sacral end plate from the anterior-posterior (AP) and superior-inferior (SI) directions. M-FHC is defined as the midpoint of both femoral head centers from the medial-lateral (ML) direction. **b** The PI angle is defined as the angle between the line perpendicular to the sacral plate at its midpoint, and the line connecting this point to the axis of the femoral heads, projected in the sagittal plane

The coordinates of the femoral head of both sides and the midpoints of the anterior and posterior edges on the sacrum were measured (Fig. 3a). Then, the midpoint of the femoral head centers (M-FHC) and the midpoint of the sacral endplate (M-SEP) was projected to the sagittal plane of the pelvis. PI was defined as the angle between a line perpendicular to the sacral plate at its midpoint and a line connecting this point to the axis of the femoral heads (Fig. 3b).

### Statistical analysis

All continuous data were normally distributed and expressed as mean ± standard deviation. The independent-sample t-test was used for comparing the differences between the PI of Crowe type I-III DDH patients and non-DDH control group. A one-way analysis of variance (ANOVA): it was used to determine whether there was a difference in the PI among the DDH patients with different Crowe types followed by post hoc testing with the Student-Newman-Keuls test. Kendall’s coefficient of concordance was used to determine the correlation between the severity of DDH and PI. The significance level (α) was set at 0.05. Statsistical analysis was performed with SPSS Version 24.0 (SPSS, IBM, Chicago, IL, USA).

In order to assess the variations of intraobserver and interobserver, two researchers (RC and MH) repeated all measurements twice in a blinded manner, and they repeated the measurements after one month. All measurements were performed after removing the identifying information of the DDH patients and non-DDH controls. Intraobserver and interobserver reliabilities of our measurements were determined by calculating the interclass correlation coefficient (ICC).

## Results

The ICC showed excellent intraobserver and interobserver reliability for the femoral head center measurements, which were 0.92–0.96 and 0.91–0.94, respectively.

Crowe type I-III DDH patients had a significantly (*p* = 0.041) higher PI than the non-DDH controls: DDH 47.6 ± 8.2°, normal 44.2 ± 8.8°. Crowe type I DDH patients had a significantly (*p* = 0.038) higher PI (48.2 ± 7.6°) than the non-DDH controls, while the PI in patients with Crowe type II or III groups and the non-DDH controls showed no significant difference: Crowe type II 49.6 ± 9.6°, *p* = 0.073; Crowe type III 44.0 ± 7.4°, *p* = 0.93 (Table 2).

There was no correlation between DDH severity (Crowe I, II or III) and PI (r = 0.091, *p* = 0.222). There were no significant differences between the PI in Crowe I, II, or III DDH patients (Crowe type I-II-III, F = 1.681, *p* = 0.618; Crowe type I-II, p = 0.618; Crowe type I-III, *p* = 0.138; Crowe type II-III, *p* = 0.087). (Table 2).

**Table 2:** Compare the PI between DDH (Crowe type I-III) group and the non-DDH control group. DDH (Crowe type I-III) group has a significantly greater PI than the non-DDH control group. Patients with Crowe type I has a significantly greater PI than the non-DDH control group

Classification	PI (°)^a^
Non-DDH Control Group	44.2 (8.8; 41.8–46.6)
DDH (Crowe Type I-III) Group	47.6 (8.2; 45.6–49.9) ^b^
Crowe Type I	48.2 (7.6; 45.2–51.2) ^c^
Crowe Type II	49.6 (9.6; 44.0–55.1)
Crowe Type III	44.0 (7.4; 39.3–48.7)

^a^ Values express mean (SD; 95%CI)

^b^ Significant differences between the DDH (Crowe type I-III) group and the non-DDH controls group at 0.05 level

^c^ Significant differences between patients with Crowe type I and the non-DDH control group at 0.05 level

## Discussion

This study found that there was a significantly higher PI in DDH patients when compared to the non-DDH controls. However, there was no relationship between DDH severity and PI, which is contrary to our hypothesis.

Increased or decreased PI is an indicator of the suboptimal spinopelvic relationship. Schwab et al. that a increased PI indicated a well-tilted pelvic orientation and a pronounced lordosis [20]. Legaye et al. showed that LL was closely related to the PI and that PI established a predictive equation of the LL [7]. Next, Schwab et al. expressed the equation simply as “LL = PI + 9°” based on measurements of non-DDH adults [21]. Imai et al. reported that PI in DDH Crowe I patients was higher than that of the non-DDH controls [16]. Our results showed that the PI in Crowe type I-III DDH patients was 3.43° higher than that of the non-DDH control group, which is in agreement with the results in the literature. The increased PI indicated the erection process of the trunk, and with an increase in LL, the sagittal balance was achieved [22]. In DDH patients, the femoral head is not entirely covered by the acetabular cup. The insufficient acetabular coverage of the femoral head causes an abnormal stress distribution on the joint contact surface [23]. Therefore, DDH patients might develop a compensatory anterior inclination of the pelvis so that an approximation of the acetabular cup for the femoral head can be achieved. Fukushima et al. showed that DDH patients with increased PI had higher sacral slope (SS), which was considered to compensate for increased anterior acetabular coverage [24]. Thus, to increase acetabular coverage, a increased PI may be overdeveloped to provide sufficient sagittal balance in DDH patients.

**Fig. 4:**
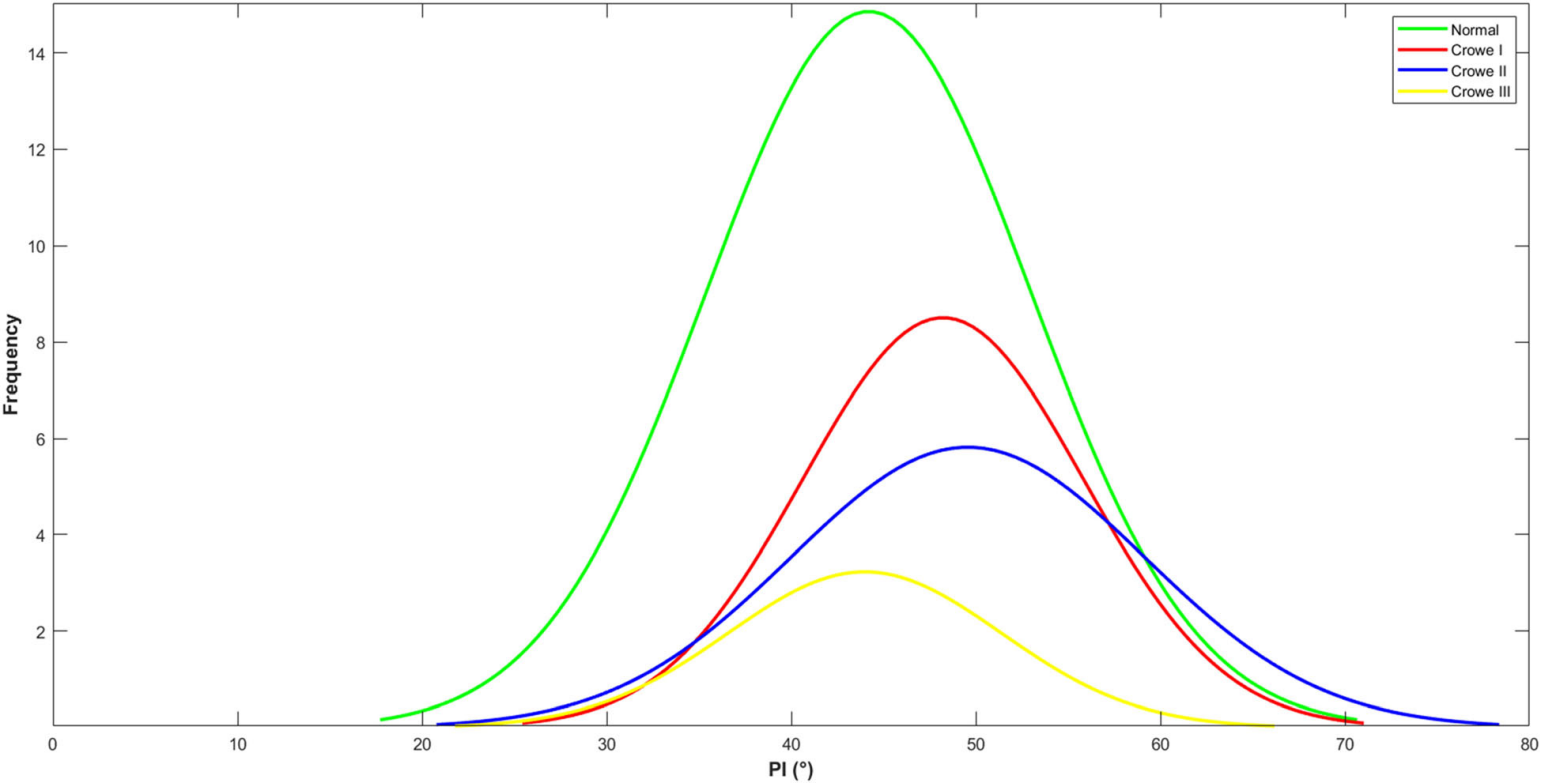
Standard deviation of the distribution of PI in Crowe I-III DDH patients and non-DDH controls

Our data showed that the PI did not correspond to the severity of the DDH. The PI in Crowe I DDH patients was significantly higher than that of the non-DDH controls, in line with others [16]. However, no significant difference was found between the non-DDH controls and DDH Crowe II (increased PI) and III (decreased PI) (Fig. 4). In other words, the compensatory anterior inclination mechanism mentioned in the last paragraph failed among Crowe III patients. We hypothesize that 75% percentage of head/socket dislocation is too hard to be compensated for, which might be one of the reasons why the PI fails to react during the development of the spinopelvic relationship. Barrey et al. analyzed the spinopelvic alignment in 85 patients with lumbar degenerative diseases [25]. They concluded that decreased PI indicated a small range of adaptation to the lumbosacral-pelvic anatomic parameters due to a small SS or a straight spine, and a small SS might lead to disc lesion. Increased PI indicated an extensive range of adaptation due to a high SS and LL and thus prone to degenerative spondylolisthesis. The conclusion by Barrey et al. explained how the sagittal balance can influence three types of degenerative pathologies, including disc herniation, degenerative disc disease involved one or two levels and degenerative spondylolisthesis [25]. Our results showed that PI was a varying parameter in Crowe type II or III DDH patients. Increased or decreased PI in DDH patients may be the cause of an elevated complications rate, such as LBP, dislocation and OA [1, 2]. Increased PI induces a higher chance of LBP due to a compensatory lumbopelvic inclination that increases mechanical stress in the lumbopelvic region [15], and the increased PI leads to a potential OA as well because of the insufficient covering of the anterior acetabulum [6, 26]. Decreased PI increased risk for post-operative THA dislocation patients [6, 26]. Therefore, the significant biomechanical alterations caused by an increased or decreased PI need to be analyzed in order to facilitate a personalized hip center positioning during THA surgery.

The PI in THA patients is associated with the determination of component position and postoperative complications including a high risk of posterior dislocation [5, 14]. Boulay et al. revealed that PI is closely related to acetabular orientation, and it is also an independent factor for predicting 3D acetabular position [14]. When the PI was decreased, inclination, and anteversion of the affected acetabulum were more pronounced in contrast to that of the normal acetabulum [14]. York et al. reported a high risk of posterior dislocation in THA patients with decreased PI [5]. Patients with THA who experienced dislocation had a lower mean PI of 45.2° than the patients without dislocation, whose mean PI was 58.6°. Our data showed that Crowe III DDH patients had a lower PI while Crowe I-II DDH patients had a higher PI than the non-DDH controls, implying personalized management should be considered. Furthermore, for DDH patients with decreased PI, hip surgeons need to pay more attention to the management of the coverage of the femoral head for hip preservative surgery and cup orientation for THA. Therefore, hip surgeon needs to increased posterior acetabular coverage to reduce complications such as the posterior dislocation.

For spine surgeons, the role of the pelvic region in sagittal balance is evident [26–28], and PI is usually taken into account. Using the SRS-Schwab Classification [29], LL should lie in the range of PI ±9°, and a PI-LL relation of greater than 10° is marked as deformity [20, 29]. In a situation of deformity, according to Murtagh et al. [30], spine surgeons can increase LL by taking away height posteriorly in the lumbar spine or by adding height anteriorly with interbody cage. However, the role of PI and its relationship with hip disorders in DDH patients may be underestimated by hip surgeons, who consider the pelvis mainly as a bone reference in implantation planning [31].

PI could be used to predict the adaptability of PT and SS regarding their changes in sitting and standing positions, which was not readily recognized by hip surgeon [14, 32]. The change of PT and SS is essential in that they determine the hip-spine relationship between different positions and that they are essential to the management of acetabular cup [33]. For example, a large PI-LL mismatch or large SS or LL loss could negatively affect the functional anteversion ranges at different positions [32]. Furthermore, Imai et al. compared the PI between women with normal hips and female patients with DDH Crowe I, and they showed that PI and anatomical-pelvic tilt were significantly greater in patients with DDH Crowe I [16]. However, in the current study, patients with Crowe I-III of both genders were studied and no correlation was found between the severity of DDH and the abnormality in PI. Combining our results with previous studies, we believe that the PI is an essential anatomical variable to be taken into consideration when performing THA among DDH patients. Therefore, we suggest surgeons using personalize treatment according to individual PI in order to minimize the complications and to assess functional biomechanics alterations.

### Limitations

Several limitations of the present study should be noted. First, the number of Crowe type II-III DDH patients was too small. Therefore, the sample size should be increased in future research. Second, as DDH in males is uncommon, the PI in men and women could not be assessed separately in this study. Third, DDH induced deformity may affect the measurement of the femoral head center. However, the ICCs were regarded as “excellent reliability” and could therefore be consistently be reproduced. Lastly, our study was limited to Chinese patients and may not be applicable to other racial or ethnic groups.

## Conclusions

The PI in patients with DDH (Crowe type I) group was significantly different from that of the non-DDH controls. However, the PI did not correlate with the severity of DDH based on the current data. We did notice that PI development among Crowe I-II and Crowe III was not a linear change, and it may be caused by different mechanisms regarding. However, the complicated relationship between PI development and the severity of DDH remains unclear. We believe SS and PT should be taken into consideration in the future. Even so, we suggest that personalized PI may be taken into account when treating DDH patients to reduce complications such as dislocations, LBP, OA.

## Data Availability

Data are available from the authors upon reasonable request and with permission of [Shanghai Ninth People’s Hospital, China].

## Abbreviations

DDH: Developmental dysplasia of the hip
OA: Osteoarthritis
LBP: Low back pain
THA: Total hip arthroplasty
PI: Pelvic incidence
LL: Lumbar lordosis
FAI: Femoroacetabular impingement
CT: Computed tomography
LCEA: Lateral center-edge angle
3D: Three-dimensional
ASIS: Anterior superior iliac spines
PSIS: Posterior superior iliac spines
PT: Pubic tubercles
APP: Anterior pelvic plane
ML: Medial-lateral
AP: Anterior-posterior
SI: Superior-inferior
M-FHC: Midpoint of the femoral head centers
M-SEP: Midpoint of the sacral endplate
ANOVA: Analysis of variance
ICC: Interclass correlation coefficient
SS: Sacral slope
R-ASIS: Right anterior superior iliac spine
L-ASIS: Left anterior superior iliac spine
MPT: Midpoint of the pubic tubercles
MAE: Midpoint of the anterior edge
MPE: Midpoint of the posterior edge
COR: Center of rotation
STD: Standard deviation
